# Development of Self-Management Indicators for Chronic Hepatitis B Patients on Antiviral Therapy: Results of a Chinese Delphi Panel Survey

**DOI:** 10.1371/journal.pone.0134125

**Published:** 2015-09-01

**Authors:** Ling-Na Kong, Ying Guo, Bo Qin, Xin Peng, Wen-Fen Zhu

**Affiliations:** 1 Department of Infectious Diseases, the First Affiliated Hospital of Chongqing Medical, University, Chongqing, PR China; 2 The Nursing College of Chongqing Medical University, Chongqing, PR China; 3 Department of Internal Medicine, Outpatient Department, the Second Affiliated Hospital of Harbin Medical University, Harbin, PR China; 4 School of Nursing, Jilin University, Changchun, PR China; University of Pavia, ITALY

## Abstract

**Objective:**

This study aimed to develop a set of indicators that could be used to measure and monitor the self-management performance for chronic hepatitis B (CHB) patients on antiviral therapy in China.

**Methods:**

A two-round Delphi study via e-mail correspondence was conducted, with a group of 30 Chinese experts. The Delphi questionnaire consisted of 53 indicators identified from a literature review. Experts rated and scored the importance of indicators on a five-point Likert scale. Consensus was considered to be reached if a median score in the top tertile (4-5) and ≥80% of panel ratings in the top tertile (4-5) after Round 2. The included indicators were validated with a group of 106 CHB patients.

**Results:**

The response rates for the first and second rounds were 90.9% (n=30) and 86.7% (n=26), respectively. Three new indicators were suggested in the first round. 55 indicators were included in the second round after modified. 45 (81.8%) indicators achieved on the level of consensus, all of which had an inter-quartile range of 1 or below. The final set included 4 domains and 45 indicators which were well accepted and understandable by CHB patients.

**Conclusion:**

This Delphi study produced a set of 45 self-management indicators for CHB patients on antiviral therapy in China. These indicators could be used to measure and monitor the patients’ self-management performance, with the goal of improving the quality of life in this population.

## Introduction

Hepatitis B virus (HBV) infection is a major global health concern. Worldwide, two billion people are currently infected with HBV, and among them 360 million are chronically infected [1]. Chronic hepatitis B (CHB) is a potentially severe form of liver disease that often progresses to cirrhosis and hepatocellular carcinoma (HCC) [2]. The primary aim of CHB treatment is to permanently suppress HBV replication to reduce the risk of development of cirrhosis, fibrosis and HCC [3,4]. There are currently two major classes of antiviral agents approved for the treatment of CHB: immunomodulatory agents (including conventional and pegylated interferon-alpha) and oral nucleotide/nucleoside analogues (NAs) [5]. However, these antiviral treatments are rarely to achieve sustained off-treatment responses [6]. As such, patients require long term, potentially lifelong therapy to derive continued clinical benefits [7].

Treatment recommendations for CHB patients often include medication adherence, regular follow-up visits, abstention from alcohol, and avoiding certain medications or foods [8–10]. However, many CHB patients may not have the skills or information required to adhere to these recommendations successfully [11]. As with other chronic diseases, there are behavioral, cognitive and social skills that can help patients participate more effectively in the disease management process [12].

Self-management is a dynamic, interactive, and daily process in which individuals engage to manage a chronic illness [13]. It usually involves actively managing and monitoring symptoms, treatment adherence, healthy lifestyle choices, coping with the psychosocial sequelae of the illness, and working in partnership with health professionals. Self-management approach has been found to promote medication adherence, self-efficacy, emotional status and quality of life in many chronic diseases [14]. The concept of self-management is important for CHB patients because CHB management primarily occurs in the home environment, not clinic settings.

HBV infection is highly endemic in China. There were about 93 million HBV carriers, and among them 30 million were patients with CHB, representing a great financial burden to patients, families, and the whole country [15]. Chinese health professionals tried to use self-management approach to improve the health and quality of life in patients with CHB. Developing indicators to measure and monitor the performance of self-management is becoming increasingly important for both patients and health professionals. Health professionals have developed self-management measurement for some chronic diseases, such as asthma, diabetes, hypertension and arthritis. There is no measurement for evaluating the status of patients’ self-management on CHB. This study aimed to use the Delphi technique to develop a set of self-management indicators to be used in CHB patients on antiviral therapy in China. These indicators are designed for measuring and monitoring the patients’ self-management performance to improve the effects of self-management program and then to improve the quality of life in this population.

## Methods

### Design

The Delphi technique was used in this study to obtain a consensus on self-management indicators for CHB patients on antiviral therapy. The Delphi technique is a methodology to obtain consensus opinions from experts on a given topic using questionnaires in a multi-stage process known as rounds [16]. According to the previous studies, two or three rounds are frequently used in the Delphi process [17]. This study involved two rounds of questionnaires to an expert panel via e-mail from May to September 2013.

A research group consisting of five researchers with backgrounds in hepatologist, infectious disease, nursing and psychology supervised and monitored the Delphi process. The group conducted the literature search, identified experts to participate the Delphi survey, prepared the questionnaire, distributed the survey, and performed data analysis.

### Ethics statement

The study did not need institutional review board approval as it did not affect patient care, and the information that it generated was used for consensual self-management indicators only. All participants were presented the objectives of the Delphi study, and provided their written consent by e-mail to the research group.

### Panel selection

A list of eligible experts was initially selected by the research group in order to ensure that they can represent all potential differences in background, occupational environment and clinical practices. According to selection criteria described by Tolsgaard et al. [18], inclusion criteria of our study were that the experts: (1) had at least 10 years’ working experience in CHB; (2) were regarded as leaders in the field of CHB practice; (3) were still actively practicing CHB care; (4) were from various geographic regions within China.

There is no consensus regarding the number of experts needed for Delphi studies. A panel size ranging from 20 to 50 was deemed appropriate [19]. In a preliminary recruitment round, 40 eligible experts were invited by e-mail to participate in the study. 33 experts agreed to participate.

### Questionnaire preparation

For CHB, self-management refers to patients’ active involvement in the daily activities they assume to control the disease and its symptoms, minimize its impact on functioning, emotions and interpersonal relationships, and cope with the disease. The Delphi questionnaire was developed by the research group. To identify potential self-management indicators, we conducted a literature search of the Medline database and China National Knowledge Infrastructure. The key words used in English and Chinese were “hepatitis B” and “self-management” or “disease management”. Furthermore, we also searched the literature about self-management measurement for other chronic diseases.

21 relevant articles were identified. A list of 53 self-management indicators was compiled on the basis of nine articles [11,20–27]. Based on the three tasks of self-management by Corbin and Straus (medical management, role management and emotional management) [28] and the domains of the already published self-management scale for other chronic disease patients [20,27,29], four major domains were identified. Treatment management (14 indicators) refers to medication adherence, symptom monitoring and management. Daily life management (17 indicators) refers to lifestyles that are conducive to the illness, such as diet, exercise and relax. Psychosocial coping (14 indicators) refers to coping styles to deal with the negative psychological and social impact brought by the illness. Information management (8 indicators) refers to ways to get more conducive information for disease treatment and control, such as communication with health professionals and other CHB patients.

The selection and wording of the indicators were the result of a discussion of the research group by referring to the wording of other maturity self-management scales. The draft of the indicators was pilot tested by three physicians and two registered nurses. Based on their feedback, the wording was adapted where needed.

### Rounds

#### First round

The first round was performed from May to July 2013. The experts received the first-round questionnaire by e-mail and they were instructed to rate and score the importance of each indicator on a five-point Likert scale (1 = very unimportant, 3 = neutral and 5 = very important). They were encouraged to give free comments on each indicator and/or to propose indicators considered as important. They were also required to provide their basic demographic information and professional characteristics. Following Round 1, median with inter-quartile range (IQR) was calculated to measure the central tendency and dispersion for the ratings, respectively. The agreement of panel ratings in the top tertile (4–5) was also calculated. Additional indicators that were proposed by more than 10% of the experts were included as new indicators in the second round [18].

#### Second round

The second round was performed from August to September 2013. The experts who had completed the first-round questionnaire were sent the second-round questionnaire by e-mail, with the results of the first round including the median, IQR and agreement of panel ratings for each indicator and their scores of the Round 1. Experts were instructed to rescore each indicator using the same five-point Likert scale with knowledge of what other experts had overall scored each indicator in the first round.

The final set of self-management indicators was based on consensus of the second round. There is no definite consensus criteria for the Delphi study, consensus levels used in previous Delphi studies ranged from 60% to 80% [30]. In this study a consensus was reached based on two selection criteria: a median score in the top tertile (4–5) and at least 80% of panel ratings in the top tertile (4–5). In addition, the top five indicators among each domain were selected as most important by analyzing the total scores of the included indicators. This procedure was performed to reveal the indicators that experts judged as priorities for CHB patients on antiviral therapy.

For both questionnaires reminders were sent in the case of non-response within three weeks. The Chinese language was used in the two rounds, but the results were subsequently translated into English.

### Feasibility test

The included indicators were used to form a self-rating scale for CHB patients on antiviral therapy. By referring other maturity self-management scales, each indicator was accompanied by a five-point Likert scale (1 = never, 2 = rarely, 3 = sometimes, 4 = often, and 5 = always). The questionnaire was validated with a group of CHB patients from two infectious diseases units to be sure that the indicators were well understandable. Patients were eligible for the survey if they were age of 18 years or older, diagnosed with CHB, were receiving antiviral therapy and volunteered to participate in the survey. The patients self-rated these indicators based on their own situations, and were asked to assess whether the number of indicators was appropriate. The response rate, the completion rate and the average completion time were calculated to test the feasibility of the questionnaire.

## Results

Of the 33 experts who agreed to participate in the study, 16 were hepatologist or infectious disease experts, and 17 were registered nurses. Among them, 30 (90.9%) completed the first-round questionnaire. Three experts did not return the first-round questionnaire and were therefore eliminated from further round. The second round was completed by 26 (26/30: 86.7%) experts. The mean age of the experts was 43.1 years (standard deviation: 7.4 years) in the first round and 42.4 years (standard deviation: 7.4 years) in the second round. The average experience in CHB care was 21.4 years in Round 1 and 20.5 years in Round 2. Geographically, participants from all six regions of Chinese mainland were included. They were from 14 hepatology or infectious diseases units. The demographic and professional characteristics information of the experts is described in Table 1.

**Table 1:** Panel characteristics.

Characteristics	Round 1 (n = 30)	Round 2 (n = 26)
**Sex**, n (%)		
Female	22 (73.3)	21 (80.8)
Male	8 (26.7)	5 (19.2)
**Age (years)**, n (%)		
30~	9 (30)	9 (34.6)
40~	15 (50)	13 (50)
50~	6 (20)	14 (15.4)
**Years of experience**, n (%)		
10~	12(40)	11 (42.3)
20~	13(43.3)	13 (50)
30~	5(16.7)	2 (7.7)
**Profession**, n (%)		
Physician	14 (46.7)	12 (46.2)
Nurse	16 (53.3)	14 (53.8)
**Department**, n (%)		
Infectious diseases	18(60)	16(61.5)
Hepatology	12(40)	10(38.5)
**Geographical Location**, n (%)		
Northeast	5 (16.7)	5 (19.2)
North China	4 (13.3)	4 (15.4)
East China	7 (23.3)	5 (19.2)
Central-southern	3 (10)	2 (7.7)
Southwest	7 (23.3)	6 (23.1)
Northwest	4 (13.4)	4 (15.4)

### First round

As shown in Table 2, of the 30 first-round experts, more than 80% gave top-tertile (4–5) ratings to 33 indicators, 70%~80% to 11 indicators, less than 70% to 9 indicators. All indicators had a median of 4 or above except two indicators (S18 and S28). Among the 53 indicators, 42 indicators (79.2%) had a high degree of consensus within the group with IQR of 1 or below. 11 indicators (20.8%) had an IQR of 1.25 or above.

**Table 2:** Results of the Delphi process.

Indicators in Round 1	Indicators in Round 2	Round1	Round1	Round 2	Round 2	Status
		Median (IQR)	% agreement (4–5)	Median (IQR)	% agreement (4–5)	
Treatment	Treatment					
1. To take prescribed medication	1. same	5(1)	100	5 (0)	100	included
2. To take medication according to instruction	2. same	5 (0)	100	5 (0)	100	included
3. To take a long term medication	3. same	5 (0)	100	5 (0)	100	included
4. To adjust medication under a doctor’s supervision	4. same	5 (0)	96.7	5 (1)	100	included
5. To monitor side effects of medication	5. same	5 (0)	96.7	5 (1)	92.3	included
6. To monitor the symptoms of CHB, e.g. fever, fatigue, jaundice, poor appetite, anorexia, upper abdominal discomfort, abdominal distension, pain of the hepatic region	6. same	5 (0.25)	93.3	5 (0)	96.2	included
7. To attend follow ups as required	7. same	5 (1)	96.7	5 (1)	100	included
8. To relieve fatigue or pain	8. same	4 (0.5)	76.7	4 (0)	88.5	included
9. To visit a doctor if any one of following symptoms appear, e.g. fever, fatigue, jaundice, anorexia, upper abdominal discomfort, abdominal distension, pain of the hepatic region	9. same	5 (1)	93.3	5 (0)	96.2	included
10. To receive standard treatment in regular hospital	10. same	5 (1)	86.7	5 (1)	96.2	included
11. Not stop medication when feeling better	11. same	5 (1)	80	5 (1)	92.3	included
12. Not stop medication for side effects	12. same	5 (1.25)	76.7	4 (1)	88.5	included
13. Not adjust amount of medication by myself	13. same	5 (1)	80	5 (1)	88.5	included
14. Not stop medication for economy	14. same	4 (2)	70	4 (2)	69.2	deleted
	^▲^15. To avoid drugs that are harmful to liver	/	/	5(1)	92.3	included
Daily life	Daily life					
15. To avoid high-fat or high-cholesterol food, e.g. fat meat, fried food, butter, cream, animal oil, yolk, pluck, caviar	^●^16. To avoid certain food, such as high-fat or high-cholesterol (e.g. fat meat, fried food, butter, cream, animal oil, yolk, pluck, caviar) and spicy foods (e.g. chilli, pepper, curry, mustard, caffeine)	4 (0)	86.7	4 (1)	88.5	included
16. To avoid spicy food, e.g. chilli, pepper, curry, mustard, caffeine	/	4 (0.5)	76.7	/	/	/
17. To avoid high-salt food, e.g. table salt, cured meat, sauces and salad dressings, cheese, pickles, instant soups, canned food, snacks	17. same	4 (1)	56.7	4(2)	73.1	deleted
18. To avoid high-calorie food, e.g. fats, oils, fried food, cream, nuts, seeds, butter, chocolate	18. same	3 (2)	43.3	3 (1)	46.2	deleted
19. To take protein-rich food, e.g. milk, fish, lean meat, eggs, beans	19. same	4 (1.25)	76.7	4 (1)	80.8	included
20. To take moderate fresh vegetables and fruits (at least 30–40% of your diet)	20. same	4 (1)	83.3	4 (1)	88.5	included
21. To have a bland diet (consisting of foods that are generally soft, low in dietary fiber, cooked rather than raw, and not spicy)	21. same	4 (2)	60	4 (2)	61.5	deleted
22. To have a balanced diet, fruit and vegetables (at least 30–40%), carbs and starchy foods (about 30%), proteins (about 20%), milk and dairy foods (about 20%), fatty and sugary foods or drinks (less than 10%)	^●^22. To keep nutrition balance, fruit and vegetables (at least 30–40%), carbs and starchy foods (about 30%),proteins (about 20%), milk and dairy foods (about 20%), fatty and sugary foods or drinks (less than 10%)	4 (2)	70	4 (0)	84.6	included
23. To control weight	23. same	4 (1)	66.7	4 (1)	69.2	deleted
24. To appropriately adjust exercise	^●^24. To adjust exercise according to symptoms	4 (1.25)	76.7	4 (1)	92.3	included
25. To exercise in an appropriate manner, e.g. walking, jogging, swimming, Tai Chi, yoga	25. same	4 (1.25)	76.7	4 (0)	84.6	included
26. To ensure adequate sleep (at least 7 or 8 hours one day)	26. same	4 (1)	86.7	4 (0)	88.5	included
27. To keep a regular life	27. same	4 (1)	83.3	4 (1)	92.3	included
28. To prevent influenza by taking necessary quarantine measures	28. same	3.5 (2)	50	4 (1)	65.4	deleted
29. To prevent transmission of HBV by taking necessary quarantine measures	29. same	4 (2)	73.3	5 (1)	84.6	included
30. To abstain from alcohol	30. same	5 (1)	96.7	5 (0)	100	included
31. To abstain from smoking	31. same	4 (1)	56.7	4 (1)	65.4	deleted
Psychosocial coping	Psychosocial coping					
32. To maintain a pleasant mood during illness	32. same	4.5 (1)	83.3	4 (0)	88.5	included
33. To be optimistic with CHB	33. same	4 (0)	86.7	4 (0)	96.2	included
34. To encourage myself when feeling depressed	34. same	4 (2)	60	4 (1)	69.2	deleted
35. To adjust mood when feeling sad or discouraged	^●^35. To make self-emotion adjustment when in negative mood	4 (1)	83.3	4 (0.25)	92.3	included
36. To express thoughts or feelings to the family	36. same	4 (1)	83.3	4 (0)	84.6	included
37. To exchange feelings with other CHB patients	37. same	4 (1)	53.3	4 (1)	69.2	deleted
38. To seek support when in difficulties in coping with CHB	38. same	4 (1)	83.3	4 (1)	88.5	included
39. To be confident with treatment effect	39. same	5 (1)	96.7	5 (1)	100	included
40. To maintain a good relationship with family or friends	40. same	4 (1)	83.3	4 (1)	84.6	included
41. To maintain normal communication with other people	41. same	4 (0)	80	4 (1)	96.2	included
42. To actively participate in group activities	^●^42. To actively participate in group activities at the physical conditions permit	4 (0.5)	76.7	4 (0)	80.8	included
43. Not worried that condition getting worse	43. same	4 (1)	73.3	4 (0)	84.6	included
44. To get understanding from family	^●^44. To get support and understanding from family	4 (1)	83.3	4 (1)	88.5	included
45. Life, work or study not influenced by CHB	45. same	4 (1)	80	4 (0.25)	84.6	included
	^▲^46. To actively respond to discrimination against CHB	/	/	4 (0.25)	84.6	included
Information	Information					
46. To keep good communication with health professionals	47. same	5 (1)	100	4 (1)	100	included
47. To consult health professionals about CHB	48. same	5 (1)	90	5 (1)	100	included
48. To discuss personal problems with health professionals	49. same	4 (1)	80	4.5 (1)	96.2	included
49. To exchange disease information with other CHB patients	50. same	4 (1)	80	4 (0)	84.6	included
50. To learn knowledge about disease from various resources	51. same	4 (1)	93.3	4 (1)	96.2	included
51. To keep the disease-related information complete	52. same	4 (1)	83.3	4 (1)	96.2	included
52. To list all the enquires to avoid anything missing when consulting health professionals	53. same	5 (1)	90	5 (1)	96.2	included
53. To make plans and goals about life to conquer the CHB	54. same	4 (1)	66.7	4 (1)	69.2	deleted
	^▲^55. To work out difficulties with health professionals	/	/	4 (0)	88.5	included

Note. Experts rated the importance of each indicator on a five-point scale where 1 = very unimportant and 5 = very important.

^▲^items added in the second round.

^●^ items revised in the second round.

IQR: inter-quartile range.

Seven indicators (S15, S16, S22, S24, S35, S42 and S44) were modified based on the experts’ comments. For example, S15 and S16 were combined into one indicator “to avoid certain food, such as high-fat, high-cholesterol and spicy foods”. New indicators were suggested by 16 of the 30 experts in the first round. Only three new indicators were suggested by more than 10% of the 30 experts and hence included in the second round. One was related to treatment management, one to psychosocial coping, and one to information management (Table 2). Thus, 55 indicators were included in the second round.

### Second round

At this step, 55 indicators were evaluated, including retained, modified, or new indicators. More than 80% of the experts gave ratings in the top tertile (4–5) to 45 indicators (45/55, 81.8%), all of which had a median of 4 or above and a high degree of consensus within the group with IQR of 1 or below. Ten indicators (10/55, 18.2%) were deleted because less than 80% of experts gave top tertile (4–5) ratings.

At the end of the process, 45 indicators were finally selected based on the second round consensus and divided under four domains: treatment management (14 indicators), daily life management (10 indicators), psychosocial coping (13 indicators) and information management (8 indicators). Descriptive statistics including the median, IQR and percentage agreement for each indicator is shown in Table 2. The top five indicators among each domain are shown in Table 3.

**Table 3:** The top five indicators among each domain.

Rank	Indicators	Total score
	Treatment	
1	2. To take medication according to instruction	128
2	1. To take prescribed medication	127
3	3. To take a long term medication	125
4	6. To monitor the symptoms of CHB	125
5	9. To visit a doctor if any one of the following symptoms appears, e.g., fever, fatigue, jaundice, poor appetite, anorexia, upper abdominal discomfort, abdominal distension, pain of the hepatic region	125
	Daily life	
1	30. To abstain from alcohol	126
2	29. To prevent transmission of HBV by taking necessary quarantine measures	114
3	27. To keep a regular life	113
4	16. To avoid certain food, such as high-fat or high-cholesterol (e.g. fat meat, fried food, butter, cream, animal oil, yolk, pluck, caviar) and spicy foods (e.g. chilli, pepper, curry, mustard, caffeine)	110
5	24. To adjust exercise according to symptoms	109
	Psychosocial coping	
1	39. To be confident with treatment effect	119
2	38. To seek support when in difficulties in coping with CHB	113
3	41. To maintain normal communication with other people	111
4	44. To get support and understanding from family	111
5	35. To make self-emotion adjustment when in negative mood	108
	Information	
1	48. To consult health professionals about CHB	121
2	53. To list all the enquires to avoid anything missing when consulting health professionals	117
3	49. To discuss personal problems with health professionals	116
4	51. To learn knowledge about disease from various resources	115
5	47. To keep good communication with health professionals	112

### Feasibility test

Of 120 questionnaires sent out to the CHB patients, 112 were returned. The response rate was 93.3%. Six questionnaires were excluded because more than two indicators were missing, resulting in the completion rate of 94.6%. The time needed to complete the questionnaire across patients ranged from 6 to 13 minutes and the average completion time was 8.58±1.78 min. 92.5% (98/106) of these patients considered the number of indicators was appropriate. The patients represented a broad range of ages (18–69 years) and the mean age was 37.4 years (standard deviation: 11.3 years). A summary of demographic characteristics of the patients was shown in S1 Table. The scores of the 45 indicators were listed in S2 Table.

## Discussion

### Summary of main findings

Previous studies have sought to identify self-management indicators for other chronic diseases in the purpose of measuring and monitoring the self-management performance of patients and then taking the targeted intervention measures to improve their self-management skills and quality of life. In this context, the present Delphi study was conducted to identify self-management indicators for CHB patients on antiviral therapy in China. Through a two-round Delphi process, 45 indicators under four domains were identified by a group of 30 Chinese experts with experience in CHB practices.

As described in detail previously, self-management involved many aspects, such as management of symptoms, psychosocial coping, seeking disease-related information, communication with healthcare professionals and so on [31]. The identified 14 indicators of treatment management referred to medication adherence and ways to improve medication adherence and symptoms. The top two indicators related to medication adherence, such as ‘to regulate medication with a doctor’s supervision’ and ‘to take prescribed medication’. This is consistent with the guidelines on CHB therapy which also emphasize the need for optimal adherence, with risk of drug-resistant HBV strains emerging if the virus has a medication vacation [32]. It is reported that a considerable number of CHB patients are likely to need long-term NAs therapy [4]. The experts also rated ‘to take a long term medication’ as one of the most important indicators. In addition, patients are suggested to pay attention to monitoring the symptoms of CHB and visiting the doctor if symptoms appear.

Contents of daily life management included many aspects. For CHB patients, daily life management referred to lifestyle changes to prevent or minimize symptoms and to reduce liver damage. The most common dietary strategies in the previous literature were avoiding certain foods or taking supplements [33]. In our study the most important indicator identified by experts was to eliminate alcohol from their diet. Other foods to avoid were fat or animal innards, and spicy foods. They were seen as exacerbating symptoms or having negative effects on the liver. Keeping regular life and moderate exercise (e.g., walking) were also beneficial to patients with this disease.

Compared with healthy participants, CHB patients in China frequently faced discrimination in all aspects of life and work, and experienced more psychosocial stress [34]. Psychosocial management was recognized as an important part of self-management for patients with chronic diseases. Considering the negative effects of CHB on psychosocial aspects and long-term therapy to achieve durable virologic suppression, CHB patients are suggested to find ways to deal with these problems. The experts identified 13 indicators of psychosocial coping. Many researchers believe that patients' positive expectations of their treatment favorably influence clinical outcomes. The experts rated the indicator ‘to be confident with the treatment effect’ as the most important. Other four indicators of the top five included self-regulating negative emotions, such as grief, frustration and depression, maintaining the normal social contacts and finding appropriate support from family or other resources.

The need for information regarding chronic diseases is a fundamental precursor to self-management. Some researchers suggested that successful self-management of chronic conditions required sufficient knowledge of the condition and its treatment [35]. The previous researches showed that some CHB patients reflected on the anxiety, depression and frustration of not understanding the facts of having CHB. The condition was considered more manageable when CHB patients with more knowledge about disease and treatment, such as infection route, signs and symptoms of CHB, the importance of medication adherence, lifestyle changes to reduce liver damage and so on [22]. The identified 8 indicators of information management in this study were thought to help CHB patients to learn more knowledge about the illness. The top five indicators mainly related to consulting health professionals about disease related problems and making good communication with them. In addition, patients are suggested to actively learn knowledge about disease from various resources.

The included indicators were then pilot tested with a group of 106 CHB patients. The response rate and completion rate showed that these indicators were well understandable and accepted by the CHB patients. The mean completion time of the questionnaire indicated that the number of indicators was appropriate.

### Strengths and limitations

The strengths of our study were as follows. The quality of the panel experts and their opinions on the given topic is seen as strength of the Delphi technique [36]. In this study, the presence of different geographical contexts (14 hospitals in six regions of China) and the average length of CHB care experience (20 years) suggested that our expert panel represented a broad and experienced group. Furthermore, response rate is important to the validity of the Delphi technique. The response rate of our study was satisfactory, namely 90.9% in the first round and 86.7% in the second round. This was a pleasing result as response rate was a recognised problem in Delphi study. Importantly, this meant that experts had much interest and active participation in this topic. Using electronic means was time- and cost- effective, which made it easier to complete the two- round Delphi process (distribution of questionnaires and reminder emails for completion of questionnaires), and provided easy access to the different geographical experts [37]. It also ensured that a single individual could not dominate the consensus formation and all experts had equal chance to change their opinions in the course of the process. Although providing the experts with the results of previous round may introduce some response bias, the goal of subsequent Delphi round was to challenge the experts whether to change their opinions once they had known the average responses of the panel.

However, the results of this study should be interpreted cautiously due to some limitations. Firstly, although the Delphi technique is a well-accepted method for assessing opinions, it has been criticized because the number and content of questions in the questionnaire is in some extent controlled by the investigators [38]. Even though they had the freedom to make comments on the given indicators, experts were inevitably forced to follow the questions we had proposed in the first round. To accommodate for this limitation we encouraged all experts to suggest new indicators that were relevant to our topic [18]. Three new indicators were suggested by more than 10% of the experts and were ultimately selected into the final set. Considering the panel ratings in the top tertile (4–5) of the three new indicators (92.3%, 84.6% and 88.5%), we did not make the further round to evaluate them. This produced a different number of round used within the final indicators evaluated. The validation of the three new indicators need to be tested in the clinical practice. Secondly, there is no agreement on the meaning of consensus for Delphi studies, and various definitions were used in previous studies. Consensus was achieved based on two selection criteria in our study. However, lack of consensus does not imply that an indicator was invalid, but may suggest that no alternatives exist or that more plausible possibilities exist yet [39]. Lastly, this methodology is relies on the perception of experts, which may influence the implementation success for lacking of actual evidence from real implementation [40]. Future applied research is needed to confirm the validity of these indicators.

## Conclusion

To the best of our knowledge, this is the first study to use the Delphi technique to identify a set of 45 self-management indicators for CHB patients on antiviral therapy in China, which can be used for measuring and monitoring the patients’ self- management performance. The study represents a starting point for developing a self-management program for CHB patients on antiviral therapy. These indicators will continue to be assessed in the subsequent studies.

## Supporting Information

S1 Table Characteristics of the patients (n = 106). (DOC)

S2 Table The scores of the 45 indicators in the feasibility test. (DOC)

## References

[1] Shepard CW, Simard EP, Finelli L, Fiore AE, Bell BP (2006) Hepatitis B virus infection: epidemiology and vaccination. Epidemiolo Rev 28: 112–125. 10.1093/epirev/mxj009 16754644

[2] Ganem D, Prince AM (2004) Hepatitis B virus infection—natural history and clinical consequences. New Engl J Med 350: 1118–1129. 15014185 10.1056/NEJMra031087

[3] Liaw YF, Kao JH, Piratvisuth T, Chan HLY, Chien RN, Liu CJ, et al (2012) Asian-Pacific consensus statement on the management of chronic hepatitis B: a 2012 update. Hepatol Int 6: 531–561. 26201469 10.1007/s12072-012-9365-4

[4] Petersen J, Buti M (2012) Considerations for the long-term treatment of chronic hepatitis B with nucleos(t)ide analogs. Expert Rev Gastroenterol Hepatol 6: 683–693. 10.1586/egh.12.52 23237254

[5] Fung J, Lai CL, Seto WK, Yuen MF (2011) Nucleoside/nucleotide analogues in the treatment of chronic hepatitis B. J Antimicrob Chemoth 66: 2715–2725. 10.1093/jac/dkr388 21965435

[6] Giang L, Selinger CP, Lee AU (2012) Evaluation of adherence to oral antiviral hepatitis B treatment using structured questionnaires. World J Hepatol 4: 43–49. 10.4254/wjh.v4.i2.43 22400085 PMC3295851

[7] Cooke GS, Main J, Thursz MR (2010) Treatment for hepatitis B. BMJ (Clinical research ed) 340: b5429. 10.1136/bmj.b5429 20051467

[8] Kamezaki H, Kanda T, Arai M, Wu S, Nakamoto S, Chiba T, et al (2013) Adherence to medication is a more important contributor to viral breakthrough in chronic hepatitis B patients treated with entecavir than in those with Lamivudine. Int J Med Sci 10: 567–574. 10.7150/ijms.5795 23533048 PMC3607242

[9] Lin CW, Lin CC, Mo LR, Chang CY, Perng DS, Hsu CC, et al (2013) Heavy alcohol consumption increases the incidence of hepatocellular carcinoma in hepatitis B virus-related cirrhosis. J Hepatol 58: 730–735. 10.1016/j.jhep.2012.11.045 23220252

[10] Yang YF, Yang LC, Yin LH (2009) Investigation on Compliance of Life Style in Patients with Chronic Hepatitis B. Nurs J Chin PLA 26: 27–29.

[11] Groessl EJ, Weingart KR, Kaplan RM, Clark JA, Gifford AL, Ho SB (2008) Living with hepatitis C: qualitative interviews with hepatitis C-infected veterans. J Gen Intern Med 23: 1959–1965. 10.1007/s11606-008-0790-y 18807097 PMC2596521

[12] Groessl EJ, Weingart KR, Stepnowsky CJ, Gifford AL, Asch SM, Ho SB (2011) The hepatitis C self-management programme: a randomized controlled trial. J Viral Hepat 18: 358–368. 10.1111/j.1365-2893.2010.01328.x 20529203

[13] Lorig KR, Holman H (2003) Self-management education: history, definition, outcomes, and mechanisms. Ann Behav Med 26: 1–7. 12867348 10.1207/S15324796ABM2601_01

[14] Chen KH, Chen ML, Lee S, Cho HY, Weng LC (2008) Self-management behaviours for patients with chronic obstructive pulmonary disease: a qualitative study. J Adv Nurs 64: 595–604. 10.1111/j.1365-2648.2008.04821.x 19120574

[15] Lu FM, Zhuang H (2009) Management of hepatitis B in China. Chin Med J (Engl) 122: 3–4. 19187608

[16] Dalkey NC (1969) The Delphi method: an experimental study of group opinion. Santa Monica: Rand Corporation.

[17] Maarsingh OR, Dros J, van Weert HC, Schellevis FG, Bindels PJ, van der Horst HE (2009) Development of a diagnostic protocol for dizziness in elderly patients in general practice: a Delphi procedure. BMC Fam Pract 10: 12 10.1186/1471-2296-10-12 19200395 PMC2660288

[18] Tolsgaard MG, Todsen T, Sorensen JL, Ringsted C, Lorentzen T, Ottesen B, et al (2013) International multispecialty consensus on how to evaluate ultrasound competence: a Delphi consensus survey. PLoS One 8: e57687 10.1371/journal.pone.0057687 23469051 PMC3585207

[19] Aronson BD, Janke KK, Traynor AP (2012) Investigating student pharmacist perceptions of professional engagement using a modified Delphi process. Am J Pharm Educ 76: 125 10.5688/ajpe767125 23049097 PMC3448463

[20] Bonner JE, Esserman D, Evon DM (2012) Reliability and validity of a self-efficacy instrument for hepatitis C antiviral treatment regimens. J Viral Hepat 19: 316–326. 10.1111/j.1365-2893.2011.01550.x 22497810 PMC3334309

[21] Cai SF, Peng DJ, Li YC (2012) The self-management education on the influence on the health behavior and liver function of patients with chronic hepatitis B treated by Lamivudine. Chi Mod Med 19: 144–146.

[22] Fry M, Bates G (2012) The tasks of self-managing hepatitis C: the significance of disclosure. Psychol Health 27: 460–474. 10.1080/08870446.2011.592982 21736430

[23] Li HX (2008) Level of self-management in patients with chronic hepatitis B. Chin J Pracl Nurs 24: 43–44.

[24] Lin CC, Anderson RM, Chang CS, Hagerty BM, Loveland-Cherry CJ (2008) Development and testing of the Diabetes Self-management Instrument: a confirmatory analysis. Res Nurs Health 31: 370–380. 10.1002/nur.20258 18213627

[25] Shen J, Qian XY (2012) Application of self management intervention in improving treatment compliance of patients with Hepatitis B virus. Chin Nurs Sci 26: 1963–1964.

[26] Yang J (2013) Development and Validation of an Online Program for Promoting Self-Management among Korean Patients with Chronic Hepatitis B. Nurs Res Pract 2013: 702079 10.1155/2013/702079 23401760 PMC3557620

[27] Zhang C, Wang W, Li J, Cai X, Zhang H, Wang H, et al (2013) Development and validation of a COPD self-management scale. Respir Care 58: 1931–1936. 10.4187/respcare.02269 23592786

[28] Corbin J. S A (1988) Unending work and care: managing chronic illness at home. San Francisco: Jossey bass.

[29] Xu W, Lin Z, Zheng L, Wang M (2011) Development of a self-management behavior scale for gastroesophageal reflux disease and its reliability and validity. J Nurs Sci 26: 38–41.

[30] Deane KHO, Ellis-Hill C, Dekker K, Davies P, Clarke CE (2003) A Delphi Survey of Best Practice Occupational Therapy for Parkinsons Disease in the United Kingdom. Brit J Occup Ther 66: 247–254.

[31] Li H, Jiang YF, Lin CC (2014) Factors associated with self-management by people undergoing hemodialysis: a descriptive study. Int J Nurs Stud 51: 208–216. 10.1016/j.ijnurstu.2013.05.012 23768411

[32] Foundation DH (2009) Chronic Hepatitis B (CHB) Recommendations. 2nd ed Victoria: Gastroenterological Society of Australia.

[33] Stoller EP, Webster NJ, Blixen CE, McCormick RA, Perzynski AT, Kanuch SW, et al (2009) Lay management of chronic disease: a qualitative study of living with hepatitis C infection. Am J Health Behav 33: 376–390. 19182983 10.5993/ajhb.33.4.4 PMC2720158

[34] Zhao X, Zhao L, Lai Y, Jiang S, Shen X, Liu S (2013) Physiological and Subjective Responses after Psychosocial Stress in Chinese Hepatitis B Patients. Stress Health. 10.1002/smi.2525 24027046

[35] Barlow J, Wright C, Sheasby J, Turner A, Hainsworth J (2002) Self-management approaches for people with chronic conditions: a review. Patient Educ Couns 48: 177–187. 12401421 10.1016/s0738-3991(02)00032-0

[36] Powell C (2003) The Delphi technique: myths and realities. J Adv Nurs 41: 376–382. 12581103 10.1046/j.1365-2648.2003.02537.x

[37] Stevens B, McGrath P, Yamada J, Gibbins S, Beyene J, Breau L, et al (2006) Identification of pain indicators for infants at risk for neurological impairment: a Delphi consensus study. BMC pediatrics 6: 1 16457711 10.1186/1471-2431-6-1 PMC1413531

[38] Palter VN, MacRae HM, Grantcharov TP (2011) Development of an objective evaluation tool to assess technical skill in laparoscopic colorectal surgery: a Delphi methodology. Am J Surg 201: 251–259. 10.1016/j.amjsurg.2010.01.031 20832048

[39] Kleynen M, Braun SM, Bleijlevens MH, Lexis MA, Rasquin SM, Halfens J, et al (2014) Using a Delphi technique to seek consensus regarding definitions, descriptions and classification of terms related to implicit and explicit forms of motor learning. PLoS One 9: e100227 10.1371/journal.pone.0100227 24968228 PMC4072669

[40] McGinn CA, Gagnon MP, Shaw N, Sicotte C, Mathieu L, Leduc Y, et al (2012) Users' perspectives of key factors to implementing electronic health records in Canada: a Delphi study. BMC Med Inform Decis Mak 12: 105 10.1186/1472-6947-12-105 22967231 PMC3470948

