# Correction: Development of Self-Management Indicators for Chronic Hepatitis B Patients on Antiviral Therapy: Results of a Chinese Delphi Panel Survey

**DOI:** 10.1371/journal.pone.0143464

**Published:** 2015-11-16

**Authors:** The PLOS ONE Staff

There is an error in affiliation 1 for authors Ling-Na Kong and Bo Qin. Affiliation 1 should read: Department of Infectious Diseases, the First Affiliated Hospital of Chongqing Medical University, Chongqing, PR China.

The publisher apologizes for the error.
