# Supplementary material for: Development of Self-Management Indicators for Chronic Hepatitis B Patients on Antiviral Therapy: Results of a Chinese Delphi Panel Survey
Source: PLoS One. 2015 Sep 1;10(9):e0134125. doi: 10.1371/journal.pone.0134125 (PMC4556706; doi:10.1371/journal.pone.0134125)
Supplement: S1 Table — (DOC) [file pone.0134125.s001.doc]

**S1 Table. Characteristics of the patients (n=106).**

| **Characteristics** | n (%) |
| --- | --- |
| **Sex** |  |
| Female | 40 (37.7) |
| Male | 66 (62.3) |
| **Education** |  |
| Primary or below | 12 (11.4) |
| Secondary | 32 (30.2) |
| High school | 24 (22.6) |
| Higher | 38 (35.8) |
| **Marital Status** |  |
| Single | 25 (23.6) |
| Married | 73(68.8) |
| Divorced | 4 (3.8) |
| Widow | 4 (3.8) |
| **Disease duration (years)** |  |
| ≤5 | 54 (50.9) |
| 6~10 | 22 (20.8) |
| 11~20 | 18 (17.0) |
| ≥21 | 12 (11.3) |
