# Supplementary material for: Development of Self-Management Indicators for Chronic Hepatitis B Patients on Antiviral Therapy: Results of a Chinese Delphi Panel Survey
Source: PLoS One. 2015 Sep 1;10(9):e0134125. doi: 10.1371/journal.pone.0134125 (PMC4556706; doi:10.1371/journal.pone.0134125)
Supplement: S2 Table — (DOC) [file pone.0134125.s002.doc]

**S2 Table. Scores of the 45 indicators in the feasibility test.**

| **Indicator** | **Mean** | **SD** | **Indicator** | **Mean** | **SD** |
| --- | --- | --- | --- | --- | --- |
| 1 | 4.04 | 1.01 | 24 | 4.05 | 1.21 |
| 2 | 4.07 | 1.06 | 25 | 3.99 | 1.34 |
| 3 | 4.01 | 1.04 | 26 | 3.65 | 0.93 |
| 4 | 3.95 | 1.05 | 27 | 3.55 | 1.03 |
| 5 | 3.29 | 1.34 | 28 | 3.24 | 1.00 |
| 6 | 3.52 | 1.30 | 29 | 3.37 | 0.98 |
| 7 | 3.75 | 1.19 | 30 | 3.33 | 1.07 |
| 8 | 4.06 | 0.94 | 31 | 3.32 | 0.91 |
| 9 | 3.69 | 1.16 | 32 | 3.95 | 0.86 |
| 10 | 3.39 | 1.25 | 33 | 4.13 | 0.83 |
| 11 | 4.39 | 0.80 | 34 | 2.98 | 1.19 |
| 12 | 3.90 | 1.17 | 35 | 2.58 | 1.19 |
| 13 | 3.78 | 1.23 | 36 | 2.98 | 1.08 |
| 14 | 4.20 | 0.97 | 37 | 3.38 | 1.23 |
| 15 | 3.44 | 1.07 | 38 | 4.14 | 0.91 |
| 16 | 3.58 | 0.98 | 39 | 4.04 | 0.99 |
| 17 | 3.72 | 0.91 | 40 | 3.81 | 1.24 |
| 18 | 3.42 | 1.08 | 41 | 2.87 | 1.28 |
| 19 | 3.40 | 1.15 | 42 | 2.62 | 1.27 |
| 20 | 3.24 | 1.15 | 43 | 3.06 | 1.19 |
| 21 | 3.67 | 0.94 | 44 | 3.28 | 1.21 |
| 22 | 3.67 | 1.01 | 45 | 3.53 | 1.20 |
| 23 | 3.46 | 1.28 |  |  |  |

SD: standard deviation
